# Supplementary material for: Identifying Active Rather than Total Methanotrophs Inhabiting Surface Soil Is Essential for the Microbial Prospection of Gas Reservoirs
Source: Microorganisms. 2024 Feb 11;12(2):372. doi: 10.3390/microorganisms12020372 (PMC10892661; doi:10.3390/microorganisms12020372)
Supplement: Supplementary file 1 [file microorganisms-12-00372-s001.zip › Supplemental material-PG.pdf]

Supplementary material

Identifying Active Rather Than Total Methanotrophs Inhabiting Surface Soil Is Essential for the Microbial Prospection of Gas Reservoirs

Kewei Xu[1, 2, 3, *], Cheng Tao[1, 2, 3], Lei Gu[1, 2, 3], Xuying Zheng[1, 2, 3], Yuanyuan Ma[1, 2, 3],

Zhengfei Yan[4], Yongge Sun[5], Yuanfeng Cai[6], Zhongjun Jia[6, 7, *]

1. State Key Laboratory of Shale Oil and Gas Enrichment Mechanisms and Effective Development, SINOPEC, Beijing, 100083, China

2. SINOPEC Key Laboratory of Petroleum Accumulation Mechanisms, Wuxi 214126, China

3. Wuxi Research Institute of Petroleum Geology, Research Institute of Petroleum Exploration & Production, SINOPEC, Wuxi, Jiangsu 214126, China.

4. School of Biotechnology, Jiangnan University, Wuxi 214122, China

5. Department of Earth Science, Zhejiang University, Hangzhou 310027, China.

6. Institute of Soil Science, Chinese Academy of Sciences, Nanjing 210008, China

7. State Key Laboratory of Black Soils Conservation and Utilization, Chinese Academy of Sciences, Changchun 130102, China

*To whom correspondence should be addressed.

E-mail: Kewei Xu: xukw.syky@sinopec.com

Zhongjun Jia: jia@issas.ac.cn

Table S1. Percentage of MOB in total microorganisms (based on MiSeq sequencing of 16S rRNA genes) in each DNA-SIP fraction of P1 (gas well soil)

Treatment	Fraction	*Methylocystis*	*Methylosarcina*	*Methylocaldum*	*Methylomicrobium*
$^{13}CH_4$	4	**33.76**	2.32	2.20	0.14
	5	**23.39**	7.57	0.89	0.44
	6	**15.57**	4.05	0.26	0.28
	Average	24.24	4.65	1.11	0.28
$^{12}CH_4$	4	1.01	0.04	0.08	0
	5	0.32	0.04	0.04	0
	6	0.04	0.01	0	0
	Average	0.46	0.03	0.04	0
In situ		0.018	0.078	0.006	0.008

Table S2. Percentage of MOB in total microorganisms (based on MiSeq sequencing of 16S rRNA genes) in each DNA-SIP fraction of J3 (dry well soil)

Treatment	Fraction	*Methylocystis*	*Methylosarcina*	*Methylocaldum*	*Methylosinus*
$^{13}CH_4$	4	**13.26**	0	0.04	0.01
	5	**8.71**	0.01	0	0.02
	6	**5.47**	0.01	0	0.01
	Average	9.15	0.01	0.01	0.01
$^{12}CH_4$	4	0.47	0.02	0	0.01
	5	0.11	0.01	0	0
	6	0.28	0.01	0	0
	Average	0.28	0.01	0.01	0.00
